# Preliminary evaluation of near-infrared vein visualization technology in the screening of palatal blood vessels

**DOI:** 10.4317/medoral.21996

**Published:** 2017-12-24

**Authors:** Emre Yaprak, Sibel Kayaalti-Yuksek

**Affiliations:** 1 Kocaeli University, Faculty of Dentistry, Department of Periodontology

## Abstract

**Background:**

Avoidance from palatal blood vessel rupture is a major concern during the palatal soft tissue graft surgery. There is no defined chair-side and case-specific palatal blood vessel detection approach to facilitate the harvesting process. The objective of this pilot study is to assess the feasibility of a near-infrared vein visualization system in the screening process of palatal blood vessels.

**Material and Methods:**

An extraoral vein visualization device (AccuVein AV400) was applied to a total of 304 hemi-maxilla of 152 individuals by two blind examiners. The study groups were classified according to their maximum inter-incisal measurements. The distances between the coronal border of the vessel image and the mid-palatal gingival margins of the adjacent teeth were measured and in each group. The correlations among the measurements were evaluated within groups.

**Results:**

The blood vessel to the adjacent teeth measurements exhibited no statistical difference between both examiners in all subjects (*p*<0.001). Correlations between the examiners gradually increased in all groups as the mouth opening rates of the subjects were increased (*p*<0.001).

**Conclusions:**

In the current state, screening of the palatal blood vessels via near-infrared vein visualization technology seems to be not suitable for every individual due to the restrictive effect of mouth opening. However, the promising results of this preliminary study demonstrated increasing consistency between the measurements of the examiners as the inter-incisal distance increase which emphasized the need an intraoral version of the device. Considering the lack of local decision-making technology for the detection of palatal blood vessels, further studies are required for development and optimization of these systems.

** Key words:**Near-infrared vein visualization, palatal graft harvesting, surgical complications.

## Introduction

Free gingival graft and subepithelial connective tissue graft operations are common periodontal plastic surgery techniques for the management of periodontal and peri-implant soft tissue deformities. Palatal keratinized mucosa is accepted to be the major donor site due to its analogy with the gingiva with respect to tissue characteristics (1). The size and shape of harvested grafts may differ depending on the extent of recipient sites as well as anatomical conditions of donor areas (2).

Damage to the palatal neurovascular bundle (NVB) which comprises greater palatine artery, vein and nerve together may lead several complications during the surgery (3). NVB injury may result in post-operative palatal paresthesia (4). Additionally, perforation of the blood vessels may lead excessive bleeding as well. The management is a disturbing and time-consuming process which may lead completion of the procedure with poor treatment outcomes (5). The possibility of this hemorrhagic complication may limit clinician from harvesting the optimum sized grafts required for the treatment demands (6). Direct pressure to the bleeding site, topical administration of hemostatic agents, laser applications, electro-surgery and suturing were suggested to be beneficial for obtaining hemostasis to some extent (7). Considering the above mentioned complications, prevention from this injury is a crucial concern in palatal graft harvesting surgery. Numerous cadaver and radiological studies suggested “safe” distances from the adjacent teeth to palatal vessels to guide clinicians for the determination of surgical borders of palatal donor sites during the graft harvesting (3,8-11). However, considering the anatomical variations use of these morphometric reference distances is not valid for each individual (4) .

**Figure 1:**
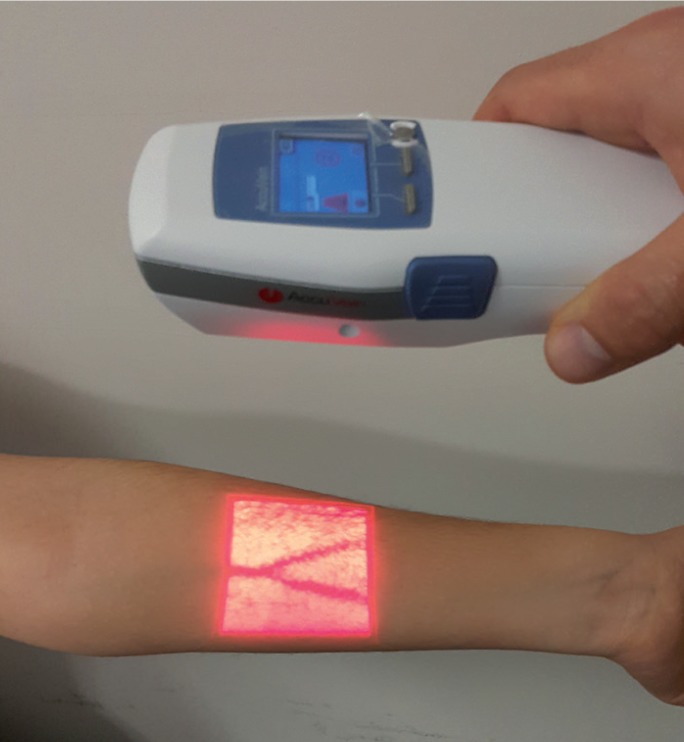
Demonstration of the VVD application for the detection of superficial veins in the arm.

**Figure 2:**
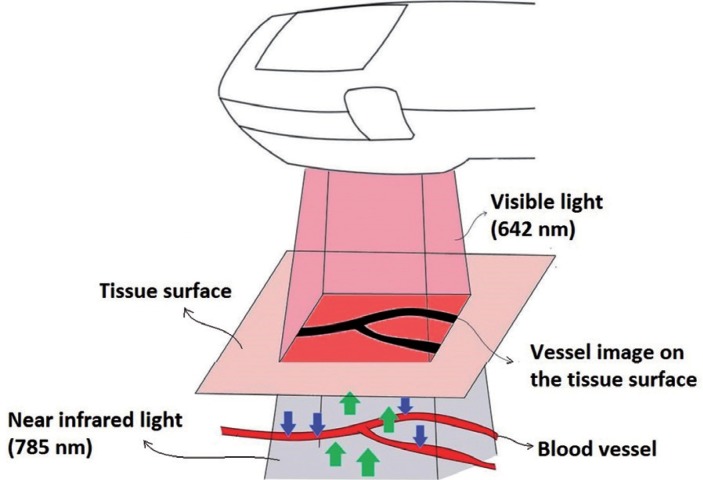
This original illustration shows the working principle of VVD. The transmitted infrared laser light (785 nm) is absorbed by hemoglobin related with its wavelength (blue arrows). Surrounding tissues other than blood vessels do not absorb the infrared light and reflect it back to the device (green arrows). An overlapping visible light (642 nm wavelength red light) is projected by the device which has the same two-dimensional contrast patterns of incoming reflected light from the tissue. Thus, the images of the blood vessel appear on the tissue surface in real-time.

Recent advances in the medical imaging devices allow real-time visualization of superficial veins under the skin (12) (Fig. 1). A laser assisted near-infrared vein visualization device (VVD) is used in medicine for screening of the vasculature on the surface of the skin (13). The device emits a near-infrared laser light (785 nm wavelength) that goes through the skin and reaches blood vessels. While projected infrared light is absorbed by hemoglobin in the blood vessels related with its wavelength, surrounding tissues other than blood vessels do not absorb infrared light and reflect it back to the device (14) (Fig. 2). A sensor in the device captures the reflected infrared light and a processor analyzes the contrast between the blood vessels and the surrounding tissues. Eventually, the device projects an overlapping visible light (642 nm wavelength), which has the same two-dimensional contrast patterns of incoming reflected light from the tissue. This allows the real-time visualization of the blood vessels on the skin surface by means of simultaneous occurrence of these optical and electronic events (15) (Fig. 2). VVDs have been used in medicine to facilitate various applications such as venipuncture, (16) obtaining vein grafts (17) and treatment of lymphatic venous anastomosis (18). This novel technology also overcomes the factors complicating vein detection with conventional approaches such as the presence of poor vein condition, dark skin color and small vein diameter with substantial superficial adipose tissue (19). Beneficial effects of VVDs especially for assisting the cases with difficult venous access have been reported to reduce unsuccessful cannulation attempts (20).

Considering those benefits of this emerging technology, intraoral use of this device to avoid palatal blood vessels can be utilized for real-time screening in palatal soft tissue graft harvesting process for the periodontal plastic surgery. To the best of our knowledge, there is no data in the literature about the use of this system for intraoral purposes and there is no defined case-specific and chair-side methodology for real-time detection of palatal blood vessels. The aim of this pilot study was to evaluate the *in vivo* use of a VVD in screening of palatal blood vessels in order to provide an initial data to test its feasibility.

## Material and Methods

- Case selection

The study was approved by the Ethics Committee of the Medical Faculty of Kocaeli University (GOKAEK 2016/55). Study group consisted of 180 voluntary staff members and students of Kocaeli University, Faculty of Dentistry. The study protocol was explained and written informed consent was obtained from all participating subjects.

All subjects who had all first and second premolar and molar teeth in their maxilla were included in the study. Third molar existence was not considered as an inclusion criteria. Individuals with a history of palatal soft tissue graft harvesting surgery, presenting any lesion or scar formation on their palatal mucosa were excluded from the study. Subjects with any acute temporomandibular joint problem or any other condition which limits their mouth opening less than 30 mm were also excluded. Inter-incisor distances of subjects at their maximal mouth opening were measured and recorded using disposable paper rulers by the same examiner (Examiner 1). A total of 28 patients were discarded due to above mentioned criteria which may interfere with VVD application process.

- VVD application and measurements

After case selection, a total of 152 individuals ( Table 1) with 304 hemi-maxillae formed the study group. Before the assessment of subjects, a calibration session was held on 10 individuals for proper application of VVD and to establish a consensus for gingival margin measurement reference points with both examiners.

**Table 1:**
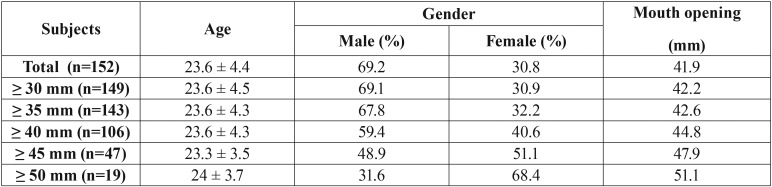
Demographic data and mouth opening values of the subjects.

Both of the examiners performed all investigation procedures independently and were unaware of one another’s results in a blind way. In this regard, all subjects were examined by the investigators within different time periods. Both of the examiners were assisted by different dental assistants who also recorded the measurements separately to evade risk of bias.

**Figure 3:**
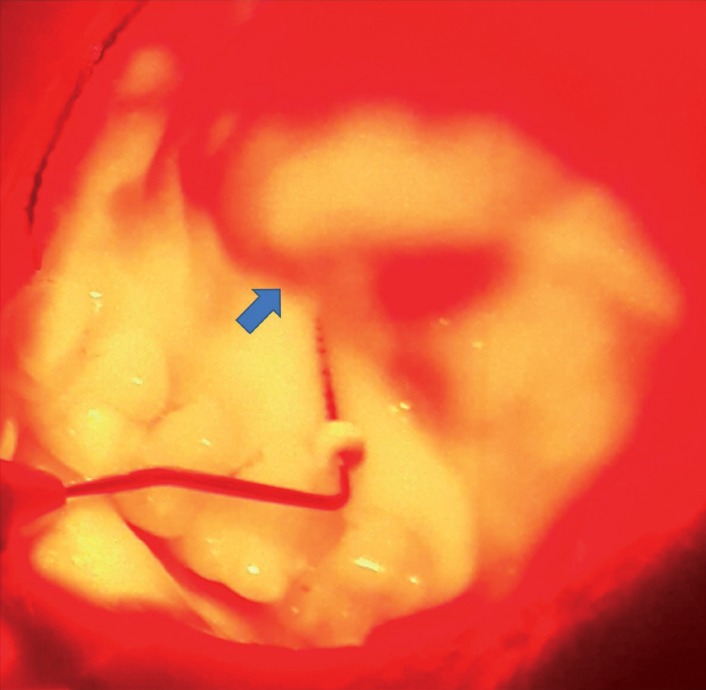
Visualization of palatal blood vessels by the VVD. The vessel image is shown with blue arrow. Please note that the vessel images were seen better with naked eye than the photographs.

AccuVeinAV400 vein visualization device (AccuVein LLC, Cold Spring Harbor, NY, US) was applied according to the manufacturer’s instructions, from a distance of 10 to 45cm over the surface and was held perpendicularly as much as possible to obtain a clear blood vessel image. Examiners and subjects wore protective glasses before the VVD application as a precaution. The device had two contrast options: i) red vessel image on a black background, ii) black vessel image on a red background. Black vessel image on a red background setting was chosen to identify the vessel image and to prevent superimposition of the mucosa color which is also red (Fig. 3). Considering black image settings, all applications were done in a dark room to facilitate vessel screening.

During the VVD application, the distances between the gingival mid-palatal margin of all upper premolars/molars and the coronal border of the vessel image were measured via rubber stopper attached Williams periodontal probes. The rubber stopper was positioned at the mid-palatal gingival margin of each tooth and the tip of the probe was located at the most coronal border of the vessel image for each measurement (Fig. 3). After adjustment of the rubber stopper on probe according to vessel localization, the distances were then measured with a digital caliper and recorded.

- Statistical analysis

Kolmogorov-Smirnov normality test was used for testing the normality of the data. Differences between the measurements of the examiners were compared using the Mann-Whitney U test at a significance level of *p*<0.05. Correlations within the examiners were analyzed with Spearman Correlation test. In order to evaluate possible effect of mouth opening rates, comparisons were also made within the groups created based on the mouth opening levels of the individuals (≥30 mm, ≥35 mm, ≥40 mm, ≥45 mm and ≥50 mm). Statistical analysis was performed with SPSS for Windows 15.0 (SPSS Inc., Chicago, IL, USA).

## Results

The study was concluded on totally 304 hemi-maxillae of 152 individuals, and demographic data and mouth opening rates of the subjects were given in Table 1. Measurements of gingival margin to coronal border of the vessel image of all subjects were presented in  Table 2 and there was no statistical difference between both examiners in all subjects (*p*<0.001). Considering the sub-groups according to the mouth opening rates; correlations between the examiners gradually increased in all groups ( Table 3) as the mouth opening rates of the subjects were increased (*p*<0.001).

**Table 2:**
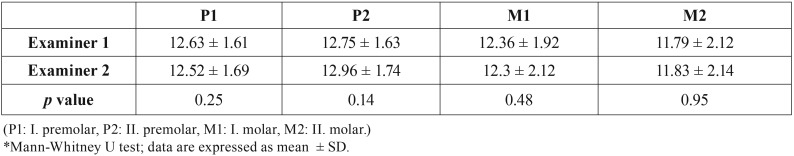
Distances (mm) measured by the examiners between palatal blood vessel image and the mid-palatal gingival margin of the teeth in all subjects (n=304).

**Table 3:**
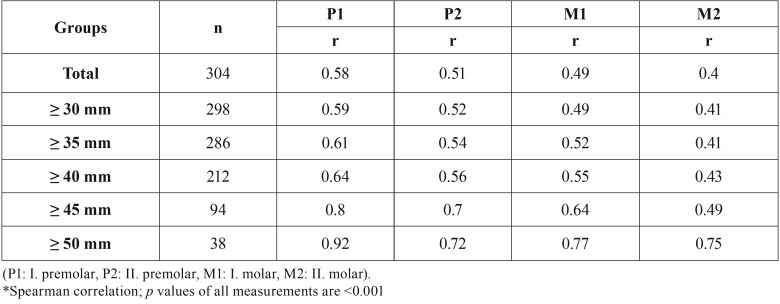
Correlation coefficients within the examiners among the groups with respect to the distances measured by the examiners.

## Discussion

Near-infrared vein visualization technology was developed for the detection of subcutaneous veins to facilitate some medical applications (14). To the best of our knowledge, there is no information about the use of VVDs for screening palatal blood vessels and there is no intraoral equipment of VVDs. Since developing, optimizing and prototyping the intraoral versions of this kind of high-tech devices obviously require some toilsome and expensive engineering processes, presence of any initial data may be valuable for the researchers to evaluate potential profits and significance before the research-development stage. On the other hand, the performance of the VVDs in the detection of palatal vessels may be affected by tissue characteristics; since the skin and palatal mucosa bear some different features (21). In addition, mouth opening ranges may restrict the proper intraoral use of these devices. In this regard, the aim of this pilot study was to provide a preliminarily data about intraoral use of an extraoral VVD for the two-dimensional detection of blood vessels in the palate and to evaluate the feasibility of this technology in the current state.

This technology is primarily based on screening of the vasculature of the skin for venipuncture purposes. However, successful detection of arteries using VVDs was also documented (22). Only hyperspectral analysis which is almost at experimental state in medicine was reported to identify veins and arteries together regarding oxygenation rate of the hemoglobin (23). For intraoral screening purposes, avoidance of all blood vessels is the primary concern rather than the discrimination of them. Additionally, both palatine arteries and veins exist together in the NVB (3). In the present study, it was not possible to differentiate whether the obtained blood vessel images belonged to an artery or a vein. Although hemorrhagic complication of an artery is severe, avoiding vein perforation is also an important concern in graft harvesting operations as well.

The vessel images are obtained within few seconds in real-time. The movements of the patient may instantly affect the quality of obtained images. Making the applications in a dark room may increase the appearance of the vessel images. In addition, red background light was sufficient to differentiate the gingival margins. There was no noticeable difference in the diameters of vessel images with respect to the application distance. Considering the acquisition of blood vessel images from all subjects included in our study, it can be speculated that intraoral use of VVD has a potential to screen the blood vessels of the palatal mucosa.

Since this is the first utilization of VVDs in oral mucosa rather than the skin, some suspicions may arise about whether the near-infrared light adequately penetrate to the palatal mucosa and reach the blood vessels. Accordingly, the thickness of palatal mucosa and the penetration capacity of this device are important issues to be addressed. The thickness of palatal mucosa has been investigated by various studies using different methodologies. From first premolar to second molar teeth; Studer et al. directly measured the thickness of palatal mucosa among the volunteers using a periodontal probe and reported the mean thicknesses as 3.9, 3.8, 3.5 and 3.5 mm (24). Using cone-beam computerized tomography technology, Barriviera et al. reported the thickness measurements as 3.93, 4.22, 4.21, 5.02 mm from the lineage of same teeth, respectively (25). In a cadaver study, Yu *et al.* investigated the thickness of palatal mucosa histologically and reported the measurements as 4.42, 4.28, 3.98, 4.85 mm (26). All those measurements were reported as the higher thickness values of which were measured at the 12 mm distance to the gingival margin. When we consider the above mentioned results of those 3 studies, the thickest part of the mucosa in the potential palatal donor sites did not exceed 5.02 mm. At this point, the penetration capacity of 785 nm laser light of VDD to the whole thickness of palatal mucosa is a critical issue and has not been documented in the literature. The efficient penetration depth of AV400 device has been reported as 8-10 mm for the skin in the literature (13). Additionally, Bashkatov *et al.* reported the optical penetration depth of 785 nm light as approximately 6 mm for mucous membranes (27). Considering the reported thickest part of palatal mucosa as 5.02 mm and reported data about penetration depth capacities of this device, it can be speculated that its effectiveness seems to be plausible in the palate. Further in vitro studies may be required to reveal exact penetration capacity of VVDs for the palatal mucosa.

According to the results, there was no statistical difference between the measurements of the examiners. The correlations among the examiners were substantially effected by the mouth opening capacities of the subjects. As the mouth opening rates of the subjects increases, the correlations became stronger. This consequence is attributed to the argument that the restrictions in the mouth opening cause improper angulation of the infrared light on the tissue surface. The lips or opposite site of the mandible may block the laser rays depending on mouth opening level and thus limit the proper angulation. The laser light must reach to the tissue surface with a perpendicular angle to completely transmit to the tissue depths and to reach the blood vessels as much as possible. Narrow angulation of the light leads its reflection from the surface and reduces its transmission and absorption rate by the vessels. In addition, as the angle of laser beams from the tissue surface decreases, the reflected rays do not return within the same direction to the device. Thus, the device cannot notice the contrast between the vessels and surrounding tissue sufficiently. Consequently, the final vessel image cannot properly appear on the tissue surface. The most clear image of the vessels can be obtained when the infrared light is applied at a perpendicular angle (15). Therefore, the presence of insufficient mouth opening is the most prominent limitation for the use of VVDs in the palate.

However, according to the results of this study, palatal vessels were more consistently located by the blind examiners in the subjects presenting relatively high mouth opening rates. This outcome may indicate the compatibility of the palate considering the tissue characteristics for vein visualization when the restrictive effect of mouth opening is ignored. At this point, it may be concluded that the development of smaller versions of VVDs which are compatible for intraoral placement and permit perpendicular angulation of laser light to the palate may remove restrictive effect of mouth opening and potentially provide remarkable benefits for detection of palatal vessels with high accuracy.

Due to lack of a clinical standard protocol for the detection of palatal blood vessels, we were not able to compare the VVD approach with another method. Magnetic resonance imaging (MRI) was considered by us as a more safe method to obtain vessel images as control. However, magnetic susceptibility artifacts (MSA) may complicate accurate detection/differentiation of the palatal vessels using MRI. Due to the proximity of the inspection zone to the bone, there would be high probability of improper detection of these relatively thin blood vessels due to image deformities related with MSA (28). In addition, the MRI would be very uncomfortable for the volunteers (e.g. entering a claustrophobic area). Moreover, there are some recent publications related with the side effects of the contrast agents (e.g. gadonlinium) which are used during MRI (29). Considering the cost/benefit ratio, we thought that such kind of verification efforts may be more significant during the further testing the ultimate intraoral versions/prototypes of these kind of devices.

The average distances between palatal NVB and the nearest teeth are documented by numerous studies to suggest a safe distance which may aid the clinicians in the determination of surgical borders during the graft harvesting (3,8-11). However, there are some discrepancies in these reported measurements. Among these, Cho et al. reported shorter distances between the palatal artery and the nearest teeth comparing with the previous studies (10). In a cadaver study, Fu et al. investigated the accuracy of the clinicians in predicting the location of palatal NVBs via comparing estimated locations with dissection results. According to their results, accuracy of the detection of palatal NVB by the clinicians is considerably affected by anatomical conditions of the palate and the discrepancy between the clinicians could be up to 4 mm (3). In addition, individual anatomical variations in the location of greater palatal foramen have been reported (30). In this context, there may be patient related anatomic variations in the palatal NVB orientation which have a potential to cause unexpected complications during the graft harvesting.

Overall, the use of extraoral VVDs seems not suitable for every case in the current state due to the limitation of mouth opening since the most consistent results were obtained in the volunteers with high opening levels in this study. However, according to the promising result of this study, it can be concluded that it may be worth developing and optimizing the intraoral versions of these kind of devices for the detection of palatal blood vessels more accurately in every case in future. In addition, the potential use of VVDs may provide additional surgical confidence to the clinicians especially in the treatment of medically compromised patients who have no luxury to deal with surgical complications.

## Conclusions

The purpose of this preliminary study is to test the feasibility and consistency of laser assisted vein visualization technology in two-dimensional detection of palatal blood vessels. Although mouth opening exhibited a restrictive effect, increasing correlations were observed among the examiners in locating the palatal vessels. Further studies including the development of intraoral versions of VVDs would be beneficial to evaluate the clinical utility of this approach whether it is useful to reduce complication risks due to patient related anatomical variations in palatal blood vessels as a case-specific approach.
